# Effects of changing reference values on the interpretation of spirometry for rubber workers^☆^^☆☆^

**DOI:** 10.1016/j.toxrep.2023.05.011

**Published:** 2023-05-27

**Authors:** Alex López, Vicente Benavides-Cordoba, Mauricio Palacios

**Affiliations:** a Maestría en Salud Ocupacional, Universidad del Valle, Cali, Colombia; b Farmacología, Universidad del Valle, Cali, Colombia; c Facultad de Salud, Universidad del Valle, Cali, Colombia

**Keywords:** Occupational exposure, Forced expiratory volume, Spirometry, Lung diseases, Occupational disease, Predictive value of tests, Rubber

## Abstract

**Introduction:**

The National Health and Nutrition Assessment Survey (NHANES) is being adopted for interpreting spirometry in occupational examinations. Rubber workers have an elevated risk of respiratory health issues due to industrial exposure, and changes in the equations would affect spirometry monitoring programs.

**Objective:**

To determine the differences in the use of the Knudson and NHANES III equations in nonsmoking workers in the rubber industry.

**Method:**

A cross-sectional study was conducted with 75 nonsmoking workers with occupational exposure to rubber for at least two years. The factory had engineered protection controls and provided respiratory protection to the workers. Spirometry was conducted according to Spirometry Testing in Occupational Health Programs and Standardization of Spirometry: American Thoracic Society/European Respiratory Society.

**Result:**

Spirometric prediction differences were present in the restrictive pattern assessment based on forced vital capacity (FVC), in which three individuals (4%) classified as normal according to Knudson presented restrictive disease according to NHANES III; only in the record of one participant was there restrictive disease using both equations. There was an 8% discrepancy for small airway obstruction in which six workers classified as normal using NHANES III were classified as diseased (FEF 25–75 <50%) using the Knudson equation.

**Conclusion:**

In the respiratory examination of workers exposed to rubber, the NHANES III equation is better able to detect restrictive diseases than is the Knudson equation; however, the Knudson equation is more sensitive to obstructive patterns.

## Highlights

• Previous studies have shown increases in the frequency of lung disease in workers, with synergism existing between exposure to rubber compounds and smoking.
• The NHANES III and Knudson equations have shown discrepancies in identifying pulmonary function diseases, especially in the African-American population.
• In the respiratory examination of nonsmoking workers exposed to rubber, the NHANES III equation is better able to detect restrictive diseases than the Knudson equation; however, the Knudson equation is more sensitive to obstructive patterns.

## Graphical Abstract

**Figure:**
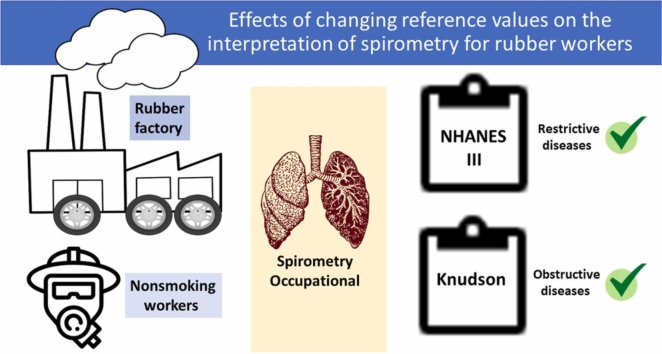

## 1 Introduction

Rubber (*caucho*) is an elastic polymer that is used in industry after vulcanization. There are two types of rubber: natural rubber that comes from the rubber tree native to South America, with production concentrated in Asia, and synthetic rubber, which is an artificial elastomer in which many polymers are synthesized from the by-products of petroleum refining; this rubber is used in approximately 70% of the rubber industry. Sixty percent of the total consumption of rubber is attributed to the production of tires, and the remainder corresponds to other products such as boots, rubber bands and rubber mulch [1].

Two compounds used to manufacture rubber, 1,3-butadiene and benzene, have been established as carcinogens in humans, and many other compounds have been reported to be associated with neoplasms. Epidemiological studies since the 1960 s have reported an increased risk of bladder cancer and leukemia, without finding differences between the use of natural and synthetic rubber [2], and have shown an association of these compounds with other types of cancer, such as stomach, lung and larynx [3], [4]. The rubber industry generates approximately 361 thousand jobs, and its impact continues to grow; it is expected that in 2018, more than 1.6 billion tires will be sold (1.3 billion were sold in 2012) [5].

Another aspect of industrial exposure to rubber corresponds to respiratory diseases in workers [6], [7]. Previous studies have shown that exposure leads to an increase in the prevalence of respiratory symptoms and in lung function diseases, especially in vital capacity (VC), forced vital capacity (FVC), forced expiratory volume in one second (FEV1) and forced expiratory flow (FEF 25–75%) and that synergism exists between exposure to rubber compounds and smoking, which increases the frequency of lung disease in these workers [8]. The diagnosis and monitoring of respiratory diseases should be evaluated by spirometry and proper interpretation. For years, different equations have been used to identify pulmonary function diseases, for example, the NHANES III and Knudson equations, but have shown discrepancies [9], especially in the African-American population [10].

However, the presentation of respiratory symptoms cannot be fully discerned by the coexistence of occupational exposure and smoking. For this reason, taking advantage of the population characteristics of smokers in Colombia, we propose to evaluate in isolation the effects of occupational exposure in the rubber industry and determine the differences in the use of the Knudson and NHANES III equations in this population. The results of this study will help define measures to prevent exposure risks and provide recommendations for the best way to monitor rubber workers.

## 2 Materials and methods

A cross-sectional study was conducted with adult Hispanic workers with occupational exposure to rubber. Workers with no history of chronic lung disease exposed to rubber manufacturing processes for at least two years were included. Workers with thoracic abnormalities that compromise adequate examination, workers with obesity (BMI > 30) that limit thoracic expansibility, workers with a history of active or passive cigarette exposure, and participants who used bronchodilators 12 h before performing the test were excluded. The population was recruited from a factory that manufactures rubber conveyor belts; the factory was engineered with measures to control particulate matter, such as extraction hoods, that allowed for controlled air exchange, conducted periodic measurements of airborne material, had an adequate ventilation system and required workers to use half facepiece respirators with filter 7093. Worker exposure was similar because they worked in the same open space. The research was approved and overseen by the research ethics committee of Universidad del Valle and was carried out in accordance with the Declaration of Helsinki and local ethical research standards.

The subjects were weighed and measured in a bipedal position, in underwear without shoes, using a calibrated scale and a stadiometer. Body weight was measured with a mechanical scale with a capacity of 180 kg and an error of 100 g, and height was measured with an aluminum stadiometer (height rod, 198 cm; Detecto CN 339) [11]. Age was recorded by complete years. Spirometry was performed by trained technicians, using standard equipment and techniques that met the guidelines of the American Thoracic Society and the European Respiratory Society (ATS/ERS). FVC, FEV1, FEV1/FVC and FEF 25–75% were measured [12], [13].

Spirometry was performed in a single pulmonary function laboratory during 2017. Spirometry was considered "abnormal" if the test met any of the following obstructive or restrictive criteria. Abnormal obstructive spirometry was defined as a forced expiratory volume in one second and forced vital capacity (FEV1/FVC) ratio below the lower limit of normal (LLN) for the reference standard. Abnormal restrictive spirometry was defined as a FEV1/FVC ratio greater than or equal to the LLN and FVC below the LLN for the reference standard. The LLN for FEV1, FVC and the FEV1/FVC ratio were obtained directly from the literature for the NHANES III reference standard [14], the Knudson reference standard [15] was calculated using the following equation:

LLN=predicted valueof the parameter−1.645(standard error of the parameter estimate)

The results were classified as normal, obstructive pattern, restrictive pattern, and small airway obstruction. For the obstructive pattern, an FEV1/FVC ratio < 0.7 or LLN was used as the reference; stratification was based on the value obtained from FEV1 according to the severity measurement [16], for the restrictive pattern, classification and stratification were performed according to the FVC; and lastly, small airway obstruction was categorized using FEF 25–75% with a value < 60%. (Table 1).

**Table 1:** **Obstructive and restrictive patterns**.

Measurement	Obstructive pattern	Restrictive pattern
Forced vital capacity (FVC)	Decreased or normal	Decreased
Forced expiratory volume in 1 s (FEV1)	Decreased	Decreased or normal
FVE1/FVC ratio	Decreased	Normal

The equations analyzed were those developed through Knudson and NHANES III. Knudson developed equations based on a North American white population of 746 people aged 8–90 years [17], these equations were designed to be used for the detection of lung disease in cotton textile workers and were subsequently modified in 1983 for use in the African-American population, with the notable limitation of not having included the Latino population.

The NHANES III equation was obtained as part of a study of the same name, led by Professor Hankinson, in which 7429 asymptomatic respiratory nonsmokers, Caucasians, African-Americans, and Mexican-Americans with ages between 8 and 80 years of age were evaluated. This sample included 2639 Mexican-Americans (1116 men and 1523 women), a population that had not been taken into account by other authors [14].

The sociodemographic variables are presented as frequencies and percentages, and the paired t-test was used to compare the means obtained from each equation, after checking for a normal distribution with the Kolmogorov-Smirnov test. The data were processed and plotted with GraphPad 6.0 software.

## 3 Results

Of the 149 workers, 40 were excluded because they were administrative workers who were not exposed. In addition, were excluded 32 participants, two who were diagnosed with chronic pulmonary disease, 18 workers with obesity or with some thoracic disorder that restricted expansion, as were ten smokers and two former smokers. Two workers who met the inclusion criteria did not agree to participate in the study; therefore, 75 participants met the study criteria. All participants were male, likely because of type of work they do for the company. The average age was 41.7 years [22–60 years], and the average exposure time was 15.34 years [2–39.5 years] (Table 2).

**Table 2:** Characteristics of the population.

n: 75	Mean	SD	Range
Age (years)	41.72	11.02	22–60
Weight (kg)	74.4	9.23	58–100
Height (cm)	170.7	5.92	156–183
BMI (kg/m^2^)	25.36	2.45	21.2–32
Time of Exposure (Years)	15.34	10.72	2–39.5

*kg: kilograms, cm: centimeters, BMI: body mass index, T. Exposure: exposure time of workers to chemical hazards. SD: standard deviation*.

In comparisons of the two equations under study, with the Knudson equation, the average predicted FVC value was 4.37 L, and with the NHANES III equation, the average predicted FVC value was 4.77 L, showing a significant difference between the two; for FEV1, the value predicted using the Knudson equation was 3.6 L and that predicted using the NHANES III equation was 3.8 L. With regard to the predicted percentage reached, the results were 90.89% with the Knudson equation and 96.65% with the NHANES III equation. In the distal airways measured for FEF 25–75%, an average predicted value of 3.86 L was obtained with the Knudson equation, and an average predicted value of 3.63 L was obtained with the NHANES III equation. All of the predicted comparisons for the measured variables were significantly different (p < 0.001) (Table 3).

**Table 3:** Comparison of spirometric parameters. Mean (SEM).

n: 75	Knudson	NHANES III	p
FVC (L)	4.37 (0.07)	4.77 (0.05)	< 0.001
FVC (%)	110.30 (1.82)	100.20 (1.36)	< 0.001
VEF1 (L)	3.60 (0.06)	3.80 (0.05)	< 0.001
VEF1 (%)	105.20 (1.83)	99.36 (1.43)	< 0.001
FEF 25–75 (L)	3.86 (0.07)	3.63 (0.07)	< 0.001
FEF 25–75 (%)	90.89 (3.02)	96.65 (3.15)	< 0.001

*F*VC (L): Forced vital capacity, in liters, FVC (%): percentage reached of forced vital capacity, FEV1 (L) Forced expiratory volume in one second, in liters, FEV1 (%) Percentage reached of forced expiratory volume in one second, FEF 25–75 (L) Forced expiratory flow between 25% and 75%, in liters. FEF 25–75 (%) Percentage reached of forced expiratory flow between 25% and 75%. SEM: Standard error of the mean.

The comparison of the diagnostic assessment between the Knudson and NHANES III equations is presented in Table 4. It can be observed that 72% of the evaluated workers (n = 54) presented similar spirometry results (normal). This similarity in diagnostic outcome was also present in the obstructive pattern, for which 6.6% of the total rubber workers had an FEV1/FVC ratio lower than the LLN.

**Table 4:** Descriptive comparison of the patterns of pulmonary function.

		NHANES III			
		Normal	Restrictive	Obstructive	Obst. SA
Knudson	**Normal**	54 (72%)	3 (4%)	-	-
	**Restrictive**	-	1 (1.3%)	-	-
	**Obstructive**	-	-	5 (6.6%)	-
	**Obst. SA**	6 (8%)	-	-	6 (8%)

Normal: spirometry without disease, Restrictive: spirometry with disease according to FVC, Obstructive: spirometry with disease according to FEV1/FVC, FEV1, Obst. SA: Small airway obstruction according to decreasing FEF 25–75%.

The difference in diagnostic equations was seen in the restrictive pattern assessment with FVC, in which three individuals (4%) classified as normal with the Knudson equation showed restrictive lung disease with the NHANES III equation; only in the record of one participant was there restrictive disease according to both equations. Similarly, for small airway (SA) obstruction, there was an 8% discrepancy in which six workers classified as normal with the NHANES III equation were considered diseased (FEF 25–75 <50%) with the Knudson equation.

## 4 Discussion

Rubber workers are exposed to kaolin and talc dust particles and to organic solvent fumes and vapors (toluene and benzene), both of which have been associated with the onset of chronic obstructive pulmonary disease (COPD) and a reduction in total lung capacity; generating both obstructive and restrictive diseases [8].

Tobacco use combined with occupational exposure has been described in rubber workers, with summative effects on the onset and progression of pulmonary disease. Efforts were made to reduce confounding factors by having strict exclusion criteria. For this reason, to obtain more reliable data and reduce confusion, workers who had exposure to cigarettes were excluded. Colombia has a low prevalence of smoking (12.8% between 18 and 69 years) compared to countries such as the United States (20.9%) [18] or to Europe, where the prevalence of cigarette smoking by men is 39% [19], and that smoking by these workers has positive synergism, increasing the risk of chronic pulmonary diseases [8], notably, in nonsmoking workers, although to a lesser extent, there is also an increase in the incidence of chronic respiratory pathology according to the results of our study.

In the study population, it was found that slightly more than 10% had some type of chronic pulmonary disease, with 6.6% presenting with obstructive pulmonary disease and 4% with restrictive pulmonary disease; these results are comparable to previous studies that showed an increase in COPD in rubber workers [7], [8] and an increase in the prevalence of pneumoconiosis, which ultimately leads to pulmonary restriction [20]. However, it is necessary to clarify that unlike our study, these studies included both smoking and nonsmoking workers.

Discrepancies were found in the results generated from the two equations, especially in the assessment of restrictive disease using the LLN of FVC. This observation is consistent with other comparisons where it is evident that the Knudson equation is less likely to show restrictive abnormality; the Knudson equation is less exact regarding predicted values in liters and classifies some results as normal that are categorized as restrictive by the NHANES III equation (9). Comparing the Knudson and NHANES III equations, the ratio of people with altered FVC was approximately 1:3 ratio, with the NHANES III equation being the most sensitive to restrictive pulmonary function diseases in the South American population [21]. This observation is due to the measurement system and not to the effects of occupational exposure. Detection of the obstructive pattern showed a discrepancy of 100% between the two equations.

Both the Knudson and NHANES III equations have been widely studied and show a similar sensitivity in the diagnosis of obstructive disease; however, they differ in the ability to diagnose restrictive disease; the NHANES III equation is more sensitive than is the Knudson equation, and its use is recommended to quickly identify pulmonary function diseases of exposed rubber workers.

One of the limitations of the study was that it was not possible to include the female population because in Colombia, there is no demand for female labor in this line of work. There are quotas regarding the proportion of women in factories; however, generally, women are mainly involved in administrative work rather than in operational work. In future studies, it is recommended to include women to assess differences in respiratory patterns and changes during menopause that could lead to pulmonary function diseases [22].

## 5 Conclusions

Rubber industry workers without a smoking history are susceptible to both obstructive and restrictive chronic pulmonary diseases with a frequency similar to workers with a history of smoking. In the assessment of pulmonary function, the NHANES III equation has a better ability to detect restrictive diseases than does the Knudson equation; therefore, its use should be recommended by the legislation for preventive assessments and monitoring carried out by occupational health programs.

## CRediT authorship contribution statement

**Alex López:** Conceptualization, Methodology, Investigation. **Vicente Benavides-Cordoba:** Conceptualization, Methodology, Investigation, Writing – original draft. **Mauricio Palacios Gomez:** Formal analysis, Supervision, Writing – review & editing.

## Declaration of Competing Interest

The authors declare that they have no known competing financial interests or personal relationships that could have appeared to influence the work reported in this paper.

## Data Availability

Data will be made available on request.
